# Methicillin-Resistant *Staphylococcus aureus* Bloodstream Infections and Injection Drug Use, Tennessee, USA, 2015–2017

**DOI:** 10.3201/eid2603.191408

**Published:** 2020-03

**Authors:** Meghana P. Parikh, Rany Octaria, Marion A. Kainer

**Affiliations:** Tennessee Department of Health, Nashville, Tennessee, USA (M.P. Parikh, R. Octaria, M.A. Kainer);; Vanderbilt University, Nashville (R. Octaria)

**Keywords:** Methicillin-resistant Staphylococcus aureus, illicit drugs, endocarditis, hepatitis C, bacteria, Tennessee, United States, MRSA, antimicrobial resistance

## Abstract

Recently, Tennessee, USA, has seen an increase in the use of commonly injected drugs, such as heroin and fentanyl. Injection drug use (IDU) practices can lead to life-threatening methicillin-resistant *Staphylococcus aureus* (MRSA) bloodstream infections (BSIs) and other serious diseases. We matched MRSA BSIs identified through the National Healthcare Safety Network to the Tennessee Hospital Discharge Data System to characterize the prevalence, demographics, and clinical characteristics associated with IDU in this disease population. Of the 7,646 MRSA BSIs identified during 2015–2017, we found that 1,839 (24.1%) were IDU-related. IDU-related BSIs increased by 118.9%; the greatest rise occurred among emergency department–onset infections (197.4%). IDU was more often associated with white, female, 18–49-year-old, and uninsured persons (p<0.001). We found >1 additional IDU-related diagnoses in 84.2% of IDU-related BSIs. Targeted harm reduction strategies for persons at high risk of IDU are necessary to reduce MRSA BSIs in acute care settings.

## Abstract 2

Rising injection drug use associated with these infections highlights the need for targeted interventions.

Methicillin-resistant *Staphylococcus aureus* (MRSA) continues to be a prominent healthcare-associated pathogen causing illness and death (*1*,*2*). As a result of the widespread implementation of infection control practices in acute-care hospitals, nationwide decreases in hospital-onset MRSA (HO MRSA) bloodstream infections (BSIs) were seen during 2005–2012. However, recent data show that since then there has been no change in the incidence of HO MRSA BSIs (*3*). Surveillance data on MRSA BSIs from acute care hospitals in Tennessee show similar patterns in decline and stabilization of HO BSIs; however, state trends in community-onset (CO) BSIs vary greatly from reported national patterns. Nationwide estimates suggest that incidence of CO MRSA BSIs has remained stable during 2005–2016 (*3*), whereas Tennessee’s statewide surveillance showed a 37.2% increase in CO MRSA BSI events during 2011–2016 (*4*).

CO MRSA BSIs, classified as having a positive blood sample collected on or before day 3 of hospitalization or during an emergency department (ED) visit (*5*), are often associated with previous healthcare procedures and hospitalizations (*6*,*7*). Additional risk factors for CO MRSA among previously healthy persons include, but are not limited to, close contact with colonized or infected persons (*8*), shared equipment that is not cleaned between users (*9*), and skin trauma (*10*,*11*). We postulate that Tennessee’s unique epidemiology of CO MRSA BSIs might be reflective of geographic differences in injection drug use (IDU) practices associated with the opioid epidemic. These patients might have clinical manifestations and risk factors that vary from those identified in previous literature. In the 2000s, opioid use was largely associated with abuse of prescription opioids, but during the past decade, the rise in opioid use and overdose deaths has been attributed to an increase in commonly injected drugs such as heroin and fentanyl (*12**–**14*). In Tennessee, although prescription opioids are still responsible for the greatest number of opioid deaths, overdose deaths associated with synthetic opioids increased by 666% and overdose deaths associated with heroin increased by 522% during 2012–2017 (*15*).

IDU has long been identified as a risk factor for invasive MRSA infections, including skin and soft tissue infections (*16**–**18*), osteomyelitis and septic arthritis (*19**–**21*), bacteremia (*17*,*22*), and endocarditis (*18*,*23*,*24*). Data from 6 sites of the Centers for Disease Control and Prevention’s Emerging Infections Program (Division of Preparedness and Emerging Infections, National Center for Emerging and Zoonotic Infectious Diseases) have shown that persons who inject drugs are >16 times more likely to develop invasive MRSA infections than persons who do not inject drugs and that the proportion of invasive MRSA associated with IDU has risen from 4.1% in 2012 to 9.2% in 2016 (*10*).

We sought to describe the prevalence of IDU-related MRSA BSI cases in acute-care hospitals across Tennessee. In addition, we examined the demographic and clinical characteristics of IDU-related and non–IDU-related cases. With these data, we aim to inform targeted efforts to improve clinical response to high-risk MRSA BSI patients in both outpatient and inpatient settings. Furthermore, increased knowledge of the indirect impacts of the opioid epidemic is imperative for the development of policy-based prevention initiatives.

## Methods

### Data Sources

We identified MRSA BSIs using the National Healthcare Safety Network (NHSN), a nationwide reporting system through which acute care hospitals in Tennessee track laboratory-identified MRSA BSIs from inpatient (IP) units and EDs (*5*). NHSN includes details on specimen collection and facility characteristics, in addition to limited patient identifiers, such as sex, date of birth, and name (optional to report).

The Tennessee Hospital Discharge Data System (HDDS) was used to further characterize demographics and clinical characteristics of MRSA BSIs identified in NHSN. HDDS captures administrative data on patient demographics, diagnoses, and procedures performed during all IP hospitalizations and ED encounters occurring in Tennessee hospitals during January 2014–June 2018 (*25*). In that time frame, all hospital visits for MRSA BSI patients were identified by matching records on patient names, dates of birth, or medical record numbers when other identifiers were unavailable. Beginning in January 2016, all HDDS diagnosis codes were documented using the International Classification of Diseases (ICD), 10th Revision, Clinical Modification (ICD-10-CM). Prior to that, codes from ICD-10-CM or the ICD’s Ninth Revision, Clinical Modification (ICD-9-CM) were allowed in HDDS.

Our study cohort included MRSA BSIs from patients >13 years of age with onset of infection during January 2015–December 2017 and with >1 IP or ED visit to any Tennessee hospital during July 2014–June 2018, as identified in HDDS. HDDS observations were excluded if full patient names or dates of birth were missing.

### Variables

We classified a case of MRSA BSI as IDU-related if any HDDS visit in the 6 months before or after blood specimen collection contained a diagnosis code for drug use (primary or secondary). The list of ICD codes used included diagnoses for dependence, abuse, poisoning, or accidental death caused by commonly injected illicit drugs (e.g., cocaine, opioids, methamphetamine) (Appendix Table 1). These codes have been used in peer-reviewed literature to estimate IDU associated with hospitalizations for other infectious diseases, such as infective endocarditis (*23*,*24*,*26*).

In accordance with NHSN guidelines, we classified a BSI event as CO if the culture was obtained on or before hospital day 3 and as HO if obtained on hospital day 4 or later, with the admission date being day 1 (*5*). We further classified CO infections as either CO-ED or CO-IP on the basis of the patient’s location at the time of culture collection. We classified same-day cultures collected in both ED and IP locations as a single CO-ED event.

We also evaluated cases for the presence of other IDU-related diagnoses in the 6 months before or after blood collection, including hospitalization for MRSA BSI. Thus, these cases could have occurred as a part of the same or different disease process as the BSI event. IDU-related diagnosis codes included endocarditis, acute or chronic hepatitis C, osteomyelitis or septic arthritis, and skin and soft tissue infections (Appendix Table 2).

### Statistical Analysis

We evaluated differences in baseline characteristics between IDU-related and non–IDU-related BSIs using a χ^2^ or Fisher exact test for categorical variables and 2-sample *t*-test for continuous variables. We further analyzed IDU-related MRSA BSI events by onset group, using a χ^2^ or Fisher exact test for categorical variables and 1-way analysis of variance for continuous variables. We performed database linkages and statistical analyses using SAS 9.4 (SAS Institute Inc., https://www.sas.com). We defined statistical significance as p<0.05. This study was approved by the Tennessee Department of Health (TDH) Institutional Review Board (project no. 1148777-1).

## Results

After excluding patients <13 years of age at the time of culture, we identified 8,251 NHSN MRSA BSI cases from 7,076 patients during 2015–2017. Of those patients, 6,548 (92.5%) were located within HDDS. In total, the matched patients represented 7,646 MRSA BSI cases included in the study cohort. We identified only 1 BSI case per person in 87.5% of patients; the maximum number of BSI events per person over the study timeframe was 8. Tennessee state residents had 89.7% of BSIs. 

MRSA BSI cases increased 17.7% over the study period, from 2,333 cases in 2015 to 2,746 in 2017 (Figure). Most cases (57%) were CO-ED. During 2015–2017, CO-ED BSIs increased by 51.8%, as compared with decreases in CO-IP (−17.9%) and HO (−11.4%) BSIs.

**Figure:**
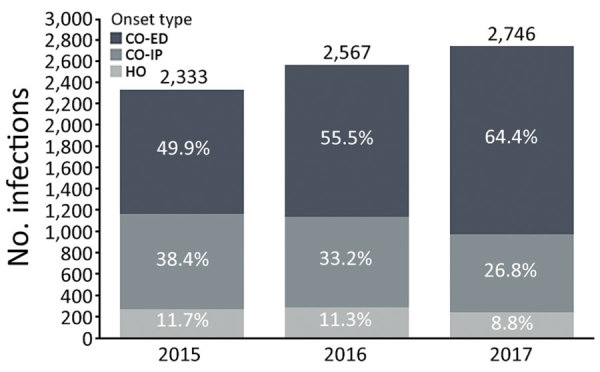
Annual cases of methicillin-resistant *Staphylococcus aureus* bloodstream infections in hospitals, stratified by onset type, Tennessee, USA, 2015–2017. CO, community onset; ED, emergency department; HO, hospital onset; IP, inpatient.

IDU-related cases represented 24.1% of the study cohort; the prevalence of these cases increased from 16.1% in 2015 to 29.9% in 2017 (Table 1). The proportion of IDU-related cases was highest among the CO-ED group (26.5%) compared with other onset groups (p<0.001). Age was associated with IDU status (p<0.001); the median age of patients with IDU-related BSIs was 40 years versus 63 years for patients with non–IDU-related BSI cases. Among IDU-related cases, 69.7% of BSIs occurred in 18–49-year-olds, whereas the same age range made up only 22.4% of non–IDU-related BSIs. Gender was also correlated with IDU status (p<0.001); men accounted for a smaller proportion of IDU-related BSIs (49.5%) than non–IDU-related BSIs (59.2%). Of all MRSA BSIs, 80.4% occurred in white patients and 17.8% in black patients. The proportion of white patients was higher among IDU-related cases than among non–IDU-related cases (88.9% versus 77.7%; p<0.001). Usage of Medicare and commercial insurance was higher among non–IDU-related BSIs (63.5% vs. 11.2% for IDU-related BSIs), whereas Medicaid usage and self-pay/uninsured status were higher among IDU-related cases (31.0% vs. 33.3% for non–IDU-related cases).

**Table 1:** Demographic and clinical characteristics of patients with methicillin-resistant *Staphylococcus aureus* bloodstream infections, by IDU status, Tennessee, USA, 2015–2017*

Characteristic	IDU status		p value	Overall
	IDU	Non-IDU		
Total	1,839 (24.1)	5,807 (75.9)	<0.001	7,646 (100)
Onset year			<0.001	
2015	375 (16.1)	1,958 (83.9)		2,333 (30.5)
2016	643 (25.0)	1,924 (75.0)		2,567 (33.6)
2017	821 (29.9)	1,925 (70.1)		2,746 (35.9)
Onset type			<0.001	
CO-ED	1,156 (62.9)	3,202 (55.1)		4,358 (57.0)
CO-IP	572 (31.1)	1,911 (32.9)		2,483 (32.5)
HO	111 (6.0)	694 (12.0)		805 (10.5)
Age range, y			<0.001	
13–17	1 (0.1)	24 (0.4)		25 (0.3)
18–34	617 (33.6)	387 (6.7)		1,004 (13.1)
35–49	665 (36.2)	912 (15.7)		1,577 (20.63)
50–64	436 (23.7)	1,789 (30.8)		2,225 (29.1)
>65	120 (6.5)	2,695 (46.4)		2,815 (36.8)
Median (range, Q1–Q3)	40 (31–51)	63 (51–74)	<0.001	
Sex			<0.001	
M	910 (49.5)	3,435 (59.2)		4,345 (56.8)
F	929 (50.5)	2,372 (40.8)		3,301 (43.2)
Race			<0.001	
White	1,635 (88.9)	4,511 (77.7)		6,146 (80.4)
Black	168 (9.1)	1,193 (20.5)		1,361 (17.8)
Other	15 (0.8)	56 (1.0)		71 (0.9)
Unknown	21 (1.1)	47 (0.8)		68 (0.9)
Ethnicity			0.820	
Hispanic	6 (0.3)	22 (0.4)		28 (0.4)
Non-Hispanic	1,768 (96.1)	5,563 (95.8)		7,331 (95.9)
Unknown	65 (3.5)	222 (3.8)		287 (3.8)
Insurance			<0.001	
Commercial	109 (5.9)	648 (11.2)		757 (9.9)
Medicaid	570 (31.0)	655 (11.3)		1,225 (16.0)
Medicare	452 (24.6)	3,688 (63.5)		4,140 (54.2)
Self-pay/uninsured	612 (33.3)	476 (8.2)		1,088 (14.2)
Other/unknown	96 (5.2)	340 (5.9)		436 (5.7)

*Values are no. (%) except as indicated. CO, community onset; ED, emergency department; HO, hospital onset; IDU, injection drug use; IP, inpatient.

Among all patients with MRSA BSIs, 4,604 (61.8%) had >1 IDU-related diagnoses documented within 6 months before or after MRSA onset. Prevalence of IDU-related diagnoses was 84.2% among patients with IDU-related BSIs and 54.7% among those with non–IDU-related BSIs. The prevalence of endocarditis (40.4%), hepatitis C infections (50.7%), osteomyelitis/septic arthritis (28.1%), and skin and soft tissue infections (46.9%) were all significantly greater (p<0.001 for all) among IDU-related BSIs than among non–IDU-related BSIs (Table 2).

**Table 2:** Prevalence of IDU-related diagnoses among patients with methicillin-resistant *Staphylococcus aureus* bloodstream infections, by IDU status, Tennessee, USA, 2015–2017*

Diagnosis	IDU, no. (%), n = 1,839	Non-IDU, no. (%), n = 5,807	p value	Overall, no. (%), n = 7,646
Endocarditis	743 (40.4)	626 (10.8)	<0.001	1,369 (17.9)
Hepatitis C	932 (50.7)	377 (6.5)	<0.001	1,309 (17.1)
Osteomyelitis/septic arthritis	516 (28.1)	1,340 (23.1)	<0.001	1,856 (24.3)
Skin/soft tissue infection	863 (46.9)	2,227 (38.4)	<0.001	3,090 (40.4)

*IDU, injection drug use.

Among IDU-related cases stratified by onset type, 62.9% were CO-ED (Table 3). The proportion of CO-ED cases increased by 18.4% during 2015–2017, whereas the proportion of CO-IP cases among IDU-related BSIs decreased by 15.5% and the proportion of HO cases among IDU-related BSIs decreased by 2.9%. CO-ED IDU-related BSIs had the youngest patients, with a median age of 38 years (p<0.001). Onset type among IDU-related BSIs was associated with insurance status (p = 0.001); the greatest usage of Medicare (37.8%) and commercial insurance (7.2%) occurred among HO cases, whereas self-pay/uninsured status was highest among CO-ED cases (36.5%). Medicaid was used most often among patients with CO-IP IDU-related BSIs (33.6%).

**Table 3:** Selected characteristics of patients with injection drug use–related methicillin-resistant *Staphylococcus aureus* bloodstream infections, by onset type, Tennessee, USA, 2015–2017*

Characteristic	MRSA onset			p value	Overall
	CO-ED	CO-IP	HO		
Total	1,156 (62.9)	572 (31.1)	111 (6.0)	<0.001	1,839 (100)
Onset year				<0.001	
2015	192 (51.2)	153 (40.8)	30 (8.0)		375 (20.4)
2016	393 (61.1)	211 (32.8)	39 (6.1)		643 (35.0)
2017	571 (69.5)	208 (25.3)	42 (5.1)		821 (44.6)
Age range, y				<0.001	
13–17	0	1 (0.2)	0		1 (0.0)
18–34	431 (37.3)	164 (28.7)	22 (19.8)		617 (33.6)
35–49	405 (35.0)	226 (39.5)	34 (30.6)		665 (36.2)
50–64	245 (21.2)	149 (26.0)	42 (37.8)		436 (23.7)
>65	75 (6.5)	32 (5.6)	13 (11.7)		120 (6.5)
Median (Q1–Q3)	38 (31–51)	42 (33–52)	49 (37–59)	<0.001	
Race				<0.001	
White	1,059 (91.6)	501 (87.6)	75 (67.6)		1,635 (88.9)
Black	83 (7.2)	56 (9.8)	29 (26.1)		168 (9.1)
Other	6 (0.5)	7 (1.2)	2 (1.8)		15 (0.8)
Unknown	8 (0.7)	8 (1.4)	5 (4.5)		21 (1.1)
Insurance				0.001	
Commercial	69 (6.0)	32 (5.6)	8 (7.2)		109 (5.9)
Medicaid	347 (30.0)	192 (33.6)	31 (27.9)		570 (31.0)
Medicare	256 (22.1)	154 (26.9)	42 (37.8)		452 (24.6)
Self-pay/uninsured	422 (36.5)	164 (28.7)	26 (23.4)		612 (33.3)
Other/unknown	62 (5.4)	30 (5.2)	4 (3.6)		96 (5.2)

*Values are no. (%) except as indicated. CO, community onset; ED, emergency department; HO, hospital onset; IP, inpatient.

## Discussion

We found an alarming increase in the extent of all MRSA BSIs in Tennessee during 2015–2017. This rise is attributed largely to the increase in the number of CO-ED cases, as CO-IP and HO cases have steadily declined. Increasing IDU over the study timeframe, as well as the high prevalence of IDU among CO-ED BSIs, suggests an association between the drug use crisis and MRSA BSIs. These trends are consistent with reports of increasing use of commonly injected drugs in Tennessee based on the surveillance of overdose deaths (*14*,*15*), which might provide an incomplete picture of current drug use practices. The use of hospital discharge billing data in our study enabled us to assess IDU among all patients entering the hospital system, including those who survived.

Using this methodology, we described common demographic characteristics of MRSA BSI patients and stratified them by IDU status. Consistent with previously reported demographics associated with IDU (*12*), we observed that IDU in our population was more common among patients who were 18–49 years of age, female, white, and uninsured. Furthermore, although still observed in the CO-IP and HO groups, IDU and those demographics were most strongly associated with ED-onset BSIs. Our findings demonstrate a shift in patient demographics typically associated with MRSA. Whereas previous studies have shown that invasive MRSA infections occur predominantly in men >49 years of age, with a larger proportion of patients being black (*27*), our study highlights an emerging at-risk population.

Currently, most public health MRSA BSI prevention and treatment strategies are targeted at HO infections (*3*,*28*). The results of this study provide a compelling argument to enhance our MRSA BSI reduction efforts by devoting resources and creating policies targeting CO BSIs. First, this new knowledge can be used to heighten awareness in ED staff of potential IDU among patients with clinical signs consistent with MRSA BSIs. These patients have a high prevalence of other IDU-related diagnoses, including endocarditis and hepatitis C, which might affect clinical progression and, ultimately, patient outcomes. Identifying patients at risk for IDU-related MRSA BSIs enables prompt diagnosis, treatment, and increased emphasis on feasible follow-up care solutions. 

A key difference between both CO groups in this study was the larger utilization of Medicaid among CO-IP IDU-related cases, compared with the higher rates of uninsurance among IDU-related CO-ED BSIs. This contrast has implications for follow-up care, because patients with IDU-related disease might be less likely to afford and pursue required follow-up treatment. In addition to the high potential for illness and death, these patients demonstrate higher rates of IDU-related infections and readmission (*29*), which are often also associated with uninsured status (*19*).

Our findings also raise a question about the role of ED and IP healthcare services in facilitating treatment for drug use and addiction. Despite evidence that interventions such as medication-assisted therapy and screening, brief intervention, and referral to treatment are both feasible and effective in acute care settings (*30**–**32*), pharmacotherapies and psychotherapies are heavily underused (*29*,*33*). Implementing interventions for substance abuse in ED settings has a large potential impact on reducing CO MRSA BSIs and other devastating consequences of IDU.

Our findings are subject to some limitations. The events included in our analyses were laboratory-identified cases from acute care hospitals sourced from Tennessee statewide surveillance data. In addition, only patients who were able to be matched to HDDS were included in the analyses; the match rate of 92.5% indicates a possible underrepresentation of the true burden of disease. We also recognize that because ICD codes do not differentiate between routes of administration for drug use, we might be overestimating the prevalence of IDU compared with overall substance abuse. Similarly, because of stigmas surrounding substance abuse, ICD codes documenting the practice might provide an underestimation of true prevalence. Although we were unable to access medical records to validate our approach, this series of diagnostic codes has previously been used to identify IDU related to infections and hospitalizations (*23*,*24*,*26*). In addition, given that hepatitis C is strongly correlated with IDU (*34*,*35*), the high prevalence of hepatitis C infections among IDU-related MRSA BSI cases in this study lends support to the validity of the diagnostic codes used. For these reasons, it is feasible that our findings are reflective not only of patterns of substance abuse, but also of IDU in Tennessee.

Our study is unique in its linkage of NHSN MRSA BSI surveillance to hospital discharge data for retrospective evaluation of IDU without conducting time-consuming chart reviews. The use of statewide laboratory-based surveillance data provides the additional benefit of a more reliable, complete picture of MRSA BSIs across Tennessee. Previous studies relied on extrapolating data from smaller jurisdictions to estimate the burden of infection and describe patient characteristics, leaving the potential for inaccurate estimation and interpretation of state trends (*10*,*27*). Our technique is advantageous for state public health agencies seeking to investigate the evolving clinical and demographic risk factors associated with reportable diseases. Despite reported national trends of unchanged CO MRSA BSIs (*3*), with the widespread nature of the opioid epidemic, we suspect that other jurisdictions, especially those with similar population characteristics as Tennessee, might see similar trends of rising CO MRSA BSIs associated with IDU. Replicating this study elsewhere would be valuable to identify any local variations in risk factors. 

In summary, Tennessee is undergoing a major change in the epidemiology of MRSA BSIs, having a growing population of young, white, uninsured, female patients with CO BSIs as a consequence of IDU. Our findings can be used to inform public health policies and clinical practice, particularly in the ED setting, to introduce prevention and harm reduction strategies to reduce the widespread impacts of this deadly disease within our communities.

Appendix Additional information on methicillin-resistant *Staphylococcus aureus* bloodstream infections and injection drug use, Tennessee, USA, 2015–2017.
