# Supplementary material for: Methicillin-Resistant Staphylococcus aureus Bloodstream Infections and Injection Drug Use, Tennessee, USA, 2015–2017
Source: Emerg Infect Dis. 2020 Mar;26(3):446–53. doi: 10.3201/eid2603.191408 (PMC7045815; doi:10.3201/eid2603.191408)
Supplement: Appendix — Additional information on methicillin-resistant Staphylococcus aureus bloodstream infections and injection drug use, Tennessee, USA, 2015–2017. [file 19-1408-Techapp-s1.pdf]

Article DOI: https://doi.org/10.3201/eid2603.191408

Methicillin-Resistant *Staphylococcus aureus* Bloodstream Infections and Injection Drug Use, Tennessee, USA, 2015–2017

Appendix

Appendix Table 1. ICD-9/10-CM codes used to identify potential injection drug use.

Diagnosis	ICD-CM codes
Opioids	ICD-9-CM: 304.0, 304.7, 305.5, 965.0, 970.1, E8500-E8502, E9350, E9352, E9401
	ICD-10-CM: F11, T400-T404, T406
Amphetamines	ICD-9-CM: 304.4, 305.7, 969.72
	ICD-10-CM: T4362
Cocaine	ICD-9-CM: 304.2, 305.6, 970.81
	ICD-10-CM: F14, T405
Other	ICD-9-CM: E8542
	ICD-10-CM: F15, F19, T507

Appendix Table 2. ICD-9/10-CM codes used to identify injection drug use-related diagnoses.

Diagnosis	ICD-CM codes
Endocarditis	ICD-9-CM: 036.42, 098.84, 112.81, 115.04, 115.14, 115.94, 421, 424.9
	ICD-10-CM: A3951, A5483, B376, I33, I38, I39
Osteomyelitis/septic arthritis	ICD-9-CM: 003.23, 003.24, 036.82, 711.0, 711.4, 711.6, 711.9, 730.0, 730.1, 730.2, 730.8, 730.9
	ICD-10-CM: A0223, A0224, A3983, M000, M001, M002, M008, M009, M01X,, M462, M463, M86, M908
Skin and/or soft tissue infection	ICD-9-CM: 040.0, 680, 681, 682, 707.1, 707.8, 707.9, 728.86, 785.4
	ICD-10-CM: A480, I96,, L02, L03, L97, L984, M726
Hepatitis C	ICD-9-CM: 070.41, 070.44, 070.51, 070.54, 070.70, 070.71, V02.62
	ICD-10-CM: B1710, B1711, B182, B1920, B1921
